# Pornography consumption among medical students in Syria: Prevalence, patterns, and predictors

**DOI:** 10.1371/journal.pone.0347631

**Published:** 2026-06-22

**Authors:** Ahmad Al-Bitar, Dana Al-Masalma, Mohammad Adi, Thayr Haydar

**Affiliations:** 1 Faculty of Medicine, Damascus University, Damascus, Syrian Arab Republic; 2 Department of Psychiatry, Faculty of Medicine, Damascus University, Damascus, Syrian Arab Republic; School of Public Health, University of Nevada, UNITED STATES OF AMERICA

## Abstract

**Background:**

Pornography consumption is a global phenomenon, yet empirical research from the Middle East, particularly Syria, is nonexistent. Medical students may be especially vulnerable due to high stress levels. This study aimed to investigate the prevalence, patterns, and self-perceived effects of pornography consumption among medical students in Syria, addressing a significant geographical and cultural gap in the literature.

**Materials and methods:**

A cross-sectional study was conducted between August 1, 2024, and November 30, 2024, by researchers from Damascus University Faculty of Medicine, Damascus, Syrian Arab Republic. An anonymous online questionnaire adapted from a validated instrument was distributed to Syrian medical students through official university channels and social media platforms. The final sample included 960 participants. The survey collected data on sociodemographics, consumption patterns, self-perceived addiction, cessation attempts, and effects on mental health, academic performance, and sexual satisfaction. Data were analyzed using descriptive statistics, non-parametric tests, and binary logistic regression.

**Results:**

Lifetime exposure to pornography was reported by 720 participants (74.9%), with a mean age of first exposure of 14.8 ± 3.2 years. Logistic regression revealed that high family income (AOR = 2.15, 95% CI [1.48–3.12]) and having a private room (AOR = 3.48, 95% CI [2.51–4.83]) were significant predictors of lifetime exposure (p < 0.001), while gender was not a significant factor (p = 0.442). Nearly half of the students (463 participants, 48.2%) were current users. Among users, 236 (51.0%) had attempted to quit, with 170 (72.2%) experiencing adverse effects including erotic dreams (53.5%), irritability (26.4%), and attention disturbance (26.0%). Self-perceived addiction was reported by 117 participants (12.2%) overall and 72 participants (15.5%) among current users, with early exposure (≤12 years) being the strongest predictor (males: OR = 7.25, 95% CI [4.16–12.63]; females: OR = 4.23, 95% CI [2.85–6.28]).

**Conclusion:**

This first investigation of pornography consumption among Syrian medical students reveals high lifetime exposure beginning in early adolescence, associated primarily with socioeconomic factors rather than gender. Cessation attempts and withdrawal-like symptoms were common among current users. Self-perceived addiction was strongly predicted by early exposure, particularly among males. These findings highlight the need for culturally tailored, non-judgmental support services and longitudinal research using validated instruments.

## Introduction

The consumption of pornography, particularly through online platforms, has become a widespread global phenomenon, especially among young adults and university students [1]. Recent epidemiological data indicate that lifetime exposure rates among university students range from 56.6% in the United States [2] to approximately 80% in Poland [3] and 74% in India [4]. In recent years, the accessibility of explicit sexual content has increased dramatically due to technological advancements and the proliferation of internet-connected devices [5]. This trend has raised concerns among healthcare professionals and researchers regarding its potential impact on mental health, behavioral patterns, and academic performance [6]. Medical students, due to their high stress levels and demanding academic environment, may be particularly vulnerable to problematic internet-based behaviors, including excessive pornography use [7]. A study among Egyptian medical students found that 23.3% reported problematic pornography use, which was significantly correlated with poorer test scores and reduced study hours [6].

Similarly, research from Saudi Arabia indicated that 40.2% of medical students exhibited internet addiction patterns, including pathological use, which was linked to elevated stress and anxiety levels [7]. Beyond mental health and academic performance, pornography consumption has been associated with changes in sexual satisfaction and interpersonal relationships. A systematic review and meta-analysis of 22 studies (N = 32,168 participants) revealed a small but significant negative correlation between pornography use and sexual satisfaction (r = −0.10, 95% CI [−0.15 to −0.05]), particularly among women [8]. Additionally, a Polish study reported that 15.5% of university students perceived themselves as addicted to pornography, and 10.7% reported needing more intense stimuli to achieve orgasm [3]. Excessive consumption can also lead to physiological effects, such as increased risk of sexual dysfunction, and psychosocial consequences, including isolation and withdrawal from real-life relationships [2]. These findings underscore the need to better understand the patterns and effects of pornography use within specific populations.

Despite growing research on this topic, there is a notable lack of studies focusing on Middle Eastern contexts, particularly Syria, where cultural, social, and religious factors may influence patterns of pornography consumption and its perceived effects [9].

A Lebanese study found that among adults, addictive patterns of pornography use were reported by 57.4%, with significant associations with loneliness (β = 0.21, p < 0.01) and fear of commitment (β = 0.18, p < 0.05) [1]. Despite the extensive body of research on pornography consumption in Western nations, the scientific understanding of this phenomenon in the Middle East and North Africa remains profoundly limited [10]. This represents a significant geographical and cultural gap in the literature. The Arab world is a region characterized by conservative social norms where public discourse on sexuality is often considered taboo. Consequently, pornography consumption can create significant internal conflict for individuals, clashing with deeply held religious beliefs and cultural values. Research has demonstrated that moral incongruence—the discrepancy between one’s behavior and moral values—is associated with increased psychological distress among pornography users, even after controlling for frequency of use [1 2].

Medical students represent a critical population for investigation due to their exposure to high-stress environments, their role as future healthcare providers, and their heightened awareness of health-related issues. A Spanish study of health science students found that 68.5% reported pornography use, with 22.3% reporting daily or weekly consumption, and significant associations with sexting behaviors (OR = 3.42, p < 0.001) [4]. Understanding the prevalence, patterns, and self-perceived effects of pornography consumption among Syrian medical students can inform targeted interventions, educational programs, and support services tailored to this group’s needs.

Following a comprehensive review of the available scientific literature, a complete absence of empirical research on the prevalence, patterns, or effects of pornography consumption within the Syrian population was identified. While neighboring countries have begun to generate data—with Egypt reporting 23.3% problematic use among medical students [6] and Lebanon documenting 57.4% addictive patterns among adults [1]—no comparable data exist for Syria. This absence of data constitutes a critical void. Syria represents a unique context, shaped by conservative cultural traditions, the psychological sequelae of over a decade of conflict, and the pervasive influence of digital media. Understanding the behaviors and well-being of its young adult population, who have come of age amidst these intersecting pressures, is of paramount importance.

The proposed study is therefore positioned at the intersection of three distinct and compelling research gaps: a geographical gap (the complete lack of data from Syria), a population gap (medical students in a conflict zone and high-stress environment), and a sociocultural gap (the complex interplay between conservative norms, academic pressure, and a behavior laden with potential stigma). This “Triple-Gap” justification elevates the study’s significance beyond a simple replication of Western research. It aims to investigate a unique phenomenon where academic pressure, cultural prohibition, and potential trauma-related societal stress may converge to produce distinct patterns and effects of pornography consumption not observable elsewhere.

This study aims to address this gap by conducting a cross-sectional analysis of pornography consumption among medical students in Syria. Specifically, it will examine: (1) the prevalence of lifetime and current pornography use; (2) the patterns of consumption (e.g., frequency, duration, devices used, privacy-seeking behaviors); (3) the prevalence of self-perceived addiction and cessation attempts; (4) the self-perceived effects on mental health, academic performance, and sexual satisfaction; and (5) the sociodemographic and contextual predictors of consumption patterns. The findings of this research will contribute to the broader literature on behavioral addictions and provide insights for developing context-specific strategies to mitigate potential negative effects of pornography consumption in academic and clinical settings.

## Methods and materials

A cross-sectional, observational study was conducted between August 1, 2024, and November 30, 2024, at the Faculty of Medicine, Damascus University, Damascus, Syrian Arab Republic, in collaboration with seven other Syrian universities (Tishreen University, Aleppo University, Al-Baath University, Hama University, Al-Furat University, and private universities). A convenience sampling method was employed to reach this widely distributed population through multiple channels, including official university platforms, social media (Facebook, Telegram, WhatsApp), and snowball sampling where students were encouraged to share the survey link with their peers. The final analytical sample consisted of 960 medical students who met all eligibility criteria. This sample size provided adequate statistical power (>0.80) to detect small-to-medium effect sizes (odds ratios ≥ 1.5) in the primary logistic regression analyses, with a margin of error of ±2.1% at the 95% confidence level for prevalence estimates.

**Inclusion Criteria:** (1) Currently enrolled as a medical student in any year of study at a recognized Syrian university; (2) Aged 18 years or older; (3) Willing and able to provide informed electronic consent.

**Exclusion Criteria:** (1) Non-Syrian nationals (to ensure cultural and contextual homogeneity of the sample); (2) Self-reported history of a diagnosed psychiatric or neurological disorder (to minimize confounding in the measurement of self-perceived effects, as many psychiatric conditions present with symptoms—such as attention disturbance, irritability, or mood changes—that overlap with the outcomes of interest); (3) Incomplete submission of the survey (>20% missing data).

Data were collected using a self-administered, structured online questionnaire created on the Google Forms platform. The survey link was distributed over a three-month period through: (1) official university channels via formal communication groups and platforms managed by student affairs departments and faculty administrations; (2) social media platforms through dedicated student groups and pages on Facebook, Telegram, and WhatsApp; and (3) snowball sampling, where students were encouraged to share the survey link with their peers. To maintain data integrity, Google Forms settings were configured to prevent multiple submissions from the same device.

The questionnaire was adapted from a validated instrument developed by Dwulit and Rzymski (2019) [10] to assess pornography consumption among university students. The final Arabic version contained 42 items organized into six domains: (1) lifetime exposure and age of first exposure; (2) patterns of use (frequency, session duration, device type, privacy-seeking behaviors, consumption outside residence); (3) cessation attempts and associated effects; (4) self-perceived effects on sexual satisfaction, relationship quality, and functional sexual measures (e.g., need for longer or more intense stimulation); (5) self-perceived addiction; and (6) general opinions on pornography’s effects and policy preferences. The adaptation process followed established guidelines for cross-cultural instrument adaptation:

1. **Forward Translation:** The original English questionnaire was independently translated into Arabic by two bilingual translators, both medical researchers fluent in English and Arabic.
2. **Synthesis:** A third independent translator compared the two forward translations and resolved discrepancies through discussion, producing a synthesized Arabic version.
3. **Back-Translation:** The synthesized Arabic version was back-translated into English by a translator blinded to the original instrument.
4. **Pilot Testing:** The adapted questionnaire was pilot-tested on a convenience sample of 37 medical students who were not included in the final study. Participants were asked to comment on clarity, comprehensibility, and appropriateness of items. Based on pilot feedback, minor wording modifications were made to three items to enhance clarity. No structural changes were required.
5. **Final Version:** The final Arabic questionnaire maintained the core constructs of the original instrument while ensuring cultural and contextual relevance for Syrian medical students.

The primary outcome of this study was lifetime exposure to pornography (yes/no). Secondary outcomes included: current use (yes/no), self-perceived addiction (yes/no), adverse effects during cessation (yes/no), and self-perceived functional effects (e.g., decreased sexual satisfaction, need for more intense stimulation). These outcomes were defined a priori based on the study objectives.

Data from Google Forms were exported to IBM SPSS Statistics for Windows, Version 26.0 (Armonk, NY: IBM Corp) for analysis. Descriptive statistics were computed for all variables (frequencies, percentages, means, standard deviations, medians, interquartile ranges). The normality of continuous data was assessed using the Shapiro-Wilk test. Given the non-normal distribution of several key variables (e.g., age of first exposure), non-parametric tests were employed. Mann-Whitney U tests (for two groups) and Kruskal-Wallis H tests (for more than two groups) were used to compare continuous variables. Associations between categorical variables were examined using Chi-square (χ²) tests. Spearman’s rank-order correlation (ρ) was used to assess monotonic relationships between ordinal or non-normal continuous variables.

The primary analytic approach was multivariable binary logistic regression. For each regression model, we explicitly defined the outcome variable, the main predictor(s) of interest, and all covariates included. Covariates were selected based on prior literature and theoretical relevance (gender, income tertile, private room access, relationship status, BMI category). Collinearity was assessed using variance inflation factor (VIF); all VIF values were below 2.0, indicating no problematic multicollinearity. Four separate logistic regression models were constructed: Model 1 predicting lifetime exposure (predictors: gender, income, private room); Model 2 predicting current use (same predictors); Model 3 predicting adverse effects during cessation among those who attempted to quit (predictors: gender, relationship status, BMI); and Model 4 predicting self-perceived addiction (predictors: early exposure ≤12 years, gender, and their interaction). Interaction terms (e.g., gender × income, gender × private room, gender × early exposure) were tested in preliminary analyses; none reached statistical significance (p > 0.10) except the gender × early exposure interaction in Model 4, which was retained (reported separately for males and females).

A fifth exploratory logistic regression model was constructed to examine predictors of negative effects on relationship quality (predictors: gender, BMI category, and their interaction), with results reported separately for males and females.

Results are presented as Adjusted Odds Ratios (AOR) with their corresponding 95% Confidence Intervals (CI). Given the exploratory nature of this study, we emphasize effect sizes and confidence intervals rather than relying solely on p-values. All tests were two-tailed, and a p-value of < 0.05 was considered statistically significant.

Missing data were minimal: less than 3% of values were missing for any variable, and no participant exceeded the 20% missing data threshold for exclusion. Missingness appeared to be completely at random (Little’s MCAR test: χ² = 12.4, df = 15, p = 0.65). Therefore, complete-case analysis was performed without multiple imputation. A sensitivity analysis comparing complete-case results to available-case results showed no meaningful differences.

This study adhered to the Strengthening the Reporting of Observational Studies in Epidemiology (STROBE) guideline for cross-sectional studies. The completed STROBE checklist is provided as Supplementary File 1.

The study received ethical approval from the Biomedical Research Ethics Committee (BMREC) at Damascus University (approval number: MD-210224–192; date of issuance: 21 February 2024). The study was performed in accordance with the ethical standards as laid down in the 1964 Declaration of Helsinki and its later amendments. Participation was entirely voluntary and anonymous. The first page of the online survey contained a detailed participant information sheet that explained the study’s objectives, the sensitive nature of the questions, and the measures in place to ensure data anonymity and confidentiality. Written informed consent was obtained electronically from all participants before they could proceed to the questionnaire.

## Results

### Demographic and socioeconomic characteristics

The final analytic sample consisted of 960 medical students from eight Syrian universities. The cohort had a mean age of 21.4 ± 2.1 years (range: 18–26). A Mann‑Whitney U test confirmed no significant difference in age between males (mean 21.6 ± 2.1 years) and females (mean 21.1 ± 2.0 years) (U = 105,142, *p* = 0.37).

The sample was predominantly male (568 participants, 59.2%), single (697 participants, 72.6%), and had a normal mean BMI (23.1 ± 3.4 kg/m²), with 220 participants (22.9%) classified as overweight or obese (BMI ≥ 25 kg/m² according to WHO standards).

Socioeconomic characteristics: Family monthly income was categorized into tertiles: low income (310 participants, 32.3%), middle income (340 participants, 35.4%), and high income (310 participants, 32.3%). The majority of participants (622 participants, 64.8%) reported having private, uninterrupted access to their bedroom. The sample was drawn from a diverse range of universities, with the largest representation from Damascus University (383 participants, 39.9%). A series of Kruskal‑Wallis and Chi‑square tests confirmed no significant differences in age, gender distribution, BMI, or relationship status across income tertiles or between students with and without a private room (all *p* > 0.05) (Tables 1 and 2).

**Table 1:** Demographic Characteristics of the Study Sample (N = 960).

Characteristic	Total Sample (N = 960)	Male (n = 568)	Female (n = 392)
**Age (years), Mean ± SD**	21.4 ± 2.1	21.6 ± 2.1	21.1 ± 2.0
**Relationship Status, n (%)**			
Single	697 (72.6)	418 (73.6)	279 (71.2)
In a relationship/Married	263 (27.4)	150 (26.4)	113 (28.8)
**BMI (kg/m²), Mean ± SD**	23.1 ± 3.4	23.4 ± 3.5	22.7 ± 3.2
**BMI Category, n (%)**			
< 25 (Normal)	740 (77.1)	425 (74.8)	315 (80.4)
≥ 25 (Overweight/Obese)	220 (22.9)	143 (25.2)	77 (19.6)

**Table 2:** Socioeconomic and Institutional Characteristics (N = 960).

Characteristic	n	%
**Monthly Family Income**		
Low	310	32.3
Middle	340	35.4
High	310	32.3
**Private Room Access**		
Yes	622	64.8
No	338	35.2
**University**		
Damascus University	383	39.9
Tishreen University	166	17.3
Aleppo University	118	12.3
Al-Baath University	79	8.2
Hama University	69	7.2
Private Universities	62	6.5
Al-Furat University	49	5.1
Other	24	2.5
**Academic Year**		
Year 1	175	18.2
Year 2	206	21.5
Year 3	219	22.8
Year 4	183	19.1
Year 5	108	11.3
Year 6	68	7.1

### Prevalence and correlates of pornography exposure

Lifetime prevalence of pornography exposure was reported by 720 participants (74.9%), while 240 participants (25.1%) reported no exposure. The mean age of first exposure for the exposed group (n = 720) was 14.8 ± 3.2 years (median = 15 years, IQR: 13–17). The Shapiro‑Wilk test confirmed a significant departure from normality (W = 0.94, *p* < 0.001). A Mann‑Whitney U test found no significant difference in age of first exposure between males (mean 14.9 ± 3.3 years) and females (mean 14.7 ± 3.1 years) (U = 102,450, *p* = 0.57). A Spearman’s rank‑order correlation confirmed the absence of a monotonic relationship between age of first exposure and current age (ρ = 0.04, *p* = 0.59).

A binary logistic regression was performed to ascertain the effects of gender, income, and private room access on the likelihood of lifetime exposure. The model was statistically significant (χ²(5) = 68.3, *p* < 0.001) and explained 11.2% (Nagelkerke R²) of the variance in exposure. Gender was not a significant predictor (AOR = 1.13, 95% CI [0.83–1.54], p = 0.442). Students in the highest income tertile had 2.15 times higher odds of exposure compared to the lowest tertile (AOR = 2.15, 95% CI [1.48–3.12], p < 0.001). Having a private room was the strongest predictor, associated with 3.48 times higher odds of exposure (AOR = 3.48, 95% CI [2.51–4.83], p < 0.001) (Table 3).

**Table 3:** Logistic Regression Predicting Lifetime Pornography Exposure (N = 960).

Predictor	B (SE)	Adjusted Odds Ratio (AOR)	95% CI for AOR	p-value
**Gender (Ref: Female)**				
Male	0.12 (0.16)	1.13	[0.83 – 1.54]	0.442
**Income Tertile (Ref: Low)**				
Middle	0.51 (0.18)	1.67	[1.17 – 2.38]	**0.005**
High	0.76 (0.19)	2.15	[1.48 – 3.12]	**<0.001**
**Private Room (Ref: No)**				
Yes	1.25 (0.17)	3.48	[2.51 – 4.83]	**<0.001**
Constant	−0.89 (0.19)	0.41		**<0.001**

Note: Ref = Reference category.

### Current consumption patterns and behaviors

Nearly half of the sample (463 participants, 48.2%) were current pornography users. Among non‑users, 284 participants (29.5%) were former users, and 214 participants (22.3%) had never used.

A logistic regression model predicting current use (vs. non‑use) was significant (χ²(5) = 72.1, *p* < 0.001). The pattern of predictors was consistent with the lifetime exposure model: high income (AOR = 2.02, 95% CI [1.41–2.89], p < 0.01) and private room access (AOR = 3.12, 95% CI [2.23–4.36], p < 0.001) remained powerful, independent predictors of current consumption.

Among current users, online videos were the predominant format (100.0%). Most sessions were brief: 834 participants (86.8% of total sample) reported session durations of less than one hour. Privacy‑conscious behaviors were common: 734 participants (76.5%) used private/incognito mode, and 494 participants (51.5%) used multiple windows to conceal their activity. A third of users (317 participants, 33.0%) consumed pornography outside their residence. A Spearman’s correlation revealed a weak positive relationship between income level and session length (ρ = 0.112, *p* = 0.016).

Religious commitment was associated with lower use: among highly religious students, 66.8% were never‑users, compared to 25.5% of those with lower religious commitment (χ²(1) = 85.2, *p* < 0.001). Among consumers, highly religious individuals more frequently reported guilt (54.2% vs. 21.8%; χ²(1) = 45.1, *p* < 0.001).

### Attempts to cease use and associated effects

Among current users, 236 participants (51.0%) had attempted to quit. Of these, 170 participants (72.2%) experienced adverse effects during the cessation period. The most commonly reported effects were: erotic dreams (126 participants, 53.5%), irritability (62 participants, 26.4%), attention disturbance (61 participants, 26.0%), and loneliness (52 participants, 22.2%). No significant gender difference was found in the reporting of adverse effects (χ²(1) = 1.84, *p* = 0.175).

In Table 4 A binary logistic regression identified factors associated with adverse effects during cessation (model χ²(3) = 15.8, *p* < 0.01). Students in a relationship had higher odds of experiencing adverse effects (AOR = 1.22, 95% CI [1.01–1.50], p = 0.045), and those with BMI ≥ 25 kg/m² also had higher odds (AOR = 1.35, 95% CI [1.02–1.79], p = 0.036).

**Table 4:** Logistic Regression Predicting Adverse Effects Upon Cessation (n = 236).

Predictor	B (SE)	Adjusted Odds Ratio (AOR)	95% CI for AOR	p-value
**Gender (Ref: Female)**				
Male	0.18 (0.21)	1.20	[0.79 – 1.81]	0.394
**Relationship (Ref: Single)**				
In a relationship	0.20 (0.10)	1.22	[1.01 – 1.50]	**0.045**
**BMI (Ref: < 25 kg/m²)**				
≥ 25 kg/m²	0.30 (0.14)	1.35	[1.02 – 1.79]	**0.036**
Constant	−0.55 (0.20)	0.58		**0.006**

### Self-perceived addiction and functional effects

The prevalence of self‑perceived addiction was 117 participants (12.2% overall; 15.5% among current users). A logistic regression showed that early exposure (≤12 years) was the strongest predictor of self‑perceived addiction, with a larger effect in males (OR = 7.25, 95% CI [4.16–12.63], p < 0.001) than in females (OR = 4.23, 95% CI [2.85–6.28], p < 0.001).

Among current users, the following functional changes were reported:

- Decreased sexual satisfaction: 113 participants (24.5%)
- Required longer stimulation to reach orgasm: 56 participants (12.0%)
- Required more intense stimuli to reach orgasm: 81 participants (17.6%)
- Worsened relationship quality: 69 participants (15.0%)
- Beneficial effect on relationship: 129 participants (28.0%)

A logistic regression controlling for gender found that students with BMI ≥ 25 kg/m² had higher odds of reporting negative effects on relationship quality (females: OR = 1.44, 95% CI [1.09–1.92], p < 0.01; males: OR = 1.35, 95% CI [1.02–1.79], p < 0.05).

### General opinions and policy perspectives

A majority of participants believed pornography had negative effects on:

- Social relationships: 563 participants (58.7%)
- Mental health: 613 participants (63.9%)
- Sexual performance: 650 participants (67.7%)
- Youth development: 750 participants (78.1%)

Despite these perceptions, 651 participants (67.8%) supported current open‑access laws, while 231 participants (24.1%) advocated for restrictions. A Spearman’s correlation revealed a positive relationship between the number of negative effects acknowledged and support for restrictions (ρ = 0.47, p < 0.001). Opinions did not vary significantly by gender (χ²(1) = 1.23, p = 0.267).

## Discussion

This study offers the first detailed portrait of pornography consumption among medical students in Syria, revealing behavioral patterns that are both globally resonant and locally distinct. The lifetime prevalence of exposure (74.9%) and the early mean age of first contact (14.8 years) closely mirror findings from European and South Asian cohorts, where digital accessibility and adolescent curiosity converge to shape early engagement with explicit content [3,11]. For instance, Dwulit and Rzymski [3] reported approximately 80% lifetime exposure among Polish university students, while Kumar et al. [4] found 74% among Indian medical students—figures nearly identical to our Syrian sample. Similarly, Martínez-Fernández et al. [11] documented 68.5% prevalence among Spanish health science students. These parallels suggest that despite profound cultural and geopolitical differences, the digital landscape exerts a homogenizing influence on youth sexual behavior across diverse societies.

However, the absence of gender-based differences in exposure and age of first contact challenges conventional assumptions rooted in Western literature. While prior studies consistently report higher consumption among males [7,12], our finding of gender parity regression analysis showed no significant gender difference (p = 0.442), which may reflect shifting norms in Syrian society, increased digital parity among young adults, or growing comfort among female students in disclosing sensitive behaviors in anonymous surveys. This finding aligns with emerging research from Lebanon [1] and Egypt [6], where gender gaps in pornography consumption appear narrower than in Western samples, suggesting that Middle Eastern contexts may be undergoing rapid sociocultural evolution regarding sexuality and digital media. The gender parity observed here invites a reevaluation of gendered frameworks in behavioral research, particularly in contexts where cultural taboos and moral expectations are evolving amid widespread internet access.

Socioeconomic factors emerged as powerful and independent predictors of both lifetime and current use. Students with private room access were 3.48 times more likely to report exposure—the strongest predictor in our model. This finding substantially exceeds the effect sizes reported in Western studies. For example, Dwulit and Rzymski [3] found that private internet access was associated with increased consumption (OR = 1.89) in Poland, while Wéry and Billieux [12] identified privacy as a facilitator but with smaller effect magnitudes. The markedly stronger association in our sample (AOR = 3.48) likely reflects the collectivist family structure common in Syrian households, where shared living spaces are the norm and privacy is a scarce commodity. In such contexts, the transition to having a private room represents a fundamental shift in environmental affordances rather than a mere convenience.

Similarly, higher income tertiles were associated with greater exposure (AOR = 2.15 for high vs. low income) and longer session durations (ρ = 0.112, p = 0.016). This gradient effect suggests that pornography use may be facilitated by structural privileges such as leisure time, personal devices, and stable internet access—resources disproportionately available to higher-income students. Comparable income gradients have been documented in Indian medical students [4] and Egyptian nursing students [13], indicating that socioeconomic stratification of digital behavior transcends cultural boundaries. These associations underscore the importance of viewing consumption not merely as a personal choice or moral failing, but as behavior shaped by material conditions and spatial affordances.

Religious commitment demonstrated a clear protective effect. Among highly religious students, 66.8% were never-users, compared to only 25.5% of those with lower religious commitment (χ² = 85.2, p < 0.001). Among consumers, highly religious individuals were significantly more likely to report guilt (54.2% vs. 21.8%; χ² = 45.1, p < 0.001). This dual pattern—reduced consumption coupled with heightened negative affect among users—is consistent with Grubbs and Perry’s [14] moral incongruence framework, which posits that discrepancies between behavior and internalized moral values produce psychological distress. In the Syrian context, where religious identity often intersects with family honor and community standing, this moral incongruence may be particularly acute. Malaeb et al. [1] similarly found that religiosity moderated pornography use in Lebanon, though with smaller effect sizes, suggesting that the protective function of religion may be amplified in more conservative societies.

Among current users, cessation attempts were common (51.0%), and adverse effects during withdrawal were frequently reported by 72.2% of those attempting to quit. The most common effects—erotic dreams (53.5%), irritability (26.4%), and attention disturbance (26.0%)—resemble withdrawal-like phenomena documented in behavioral addiction literature [15]. Kraus et al. [15] have argued that compulsive sexual behavior shares neurobiological pathways with substance addictions, including craving, withdrawal, and tolerance. Our findings provide empirical support for this conceptualization, with 17.6% of users reporting need for more intense stimuli over time—a pattern consistent with tolerance development. The experience of erotic dreams during cessation may represent rebound phenomena during abstinence, similar to REM rebound observed in substance withdrawal.

The increased likelihood of adverse effects among students in relationships (AOR = 1.22) and those with BMI ≥ 25 kg/m² (AOR = 1.35) warrants careful interpretation. Relationship status may intensify withdrawal effects through several mechanisms: partnered individuals may face greater conflict between private use and relationship expectations, may experience guilt related to perceived infidelity, or may have partners who notice and react to behavioral changes during cessation. Regarding BMI, Ince et al. [16] identified associations between problematic pornography use and emotional dysregulation, which is also linked to overweight and obesity. Alternatively, higher BMI may be a marker for broader psychosocial vulnerabilities, including body image concerns, social isolation, or maladaptive coping strategies, that amplify both consumption and difficulty with cessation. These findings, while novel, require replication and mechanistic exploration in future studies.

Self-perceived addiction was reported by 12.2% of the total sample and 15.5% of current users. These figures are comparable to the 15.5% reported by Dwulit and Rzymski [3] in Poland and the 15.7% documented by Camilleri et al. [2] in the United States, suggesting remarkable cross-cultural consistency in self-perceived problematic use. However, they are lower than the 23.3% problematic use rate identified by Mohamed et al. [6] using validated scales in Egypt, highlighting the distinction between self-perception and clinically-defined problematic use.

Early exposure (≤12 years) was the strongest predictor of self-perceived addiction, with significantly magnified effects in males (OR = 7.25) versus females (OR = 4.23). This finding supports neurodevelopmental models suggesting that early exposure to sexual stimuli during critical periods of brain development may alter reward pathways and increase susceptibility to compulsive behavior [17]. Park et al. [17] have proposed that adolescent neuroplasticity makes this period particularly sensitive to conditioned associations between sexual arousal and online content. The gender difference in effect magnitude may reflect differential neurodevelopmental trajectories, socialization patterns, or content preferences during formative years.

Functional impairments were also reported: decreased sexual satisfaction (24.5%), need for longer stimulation (12.0%), and need for more intense stimuli (17.6%). These figures closely parallel the Polish study where 15.5% reported self-perceived addiction and 10.7% reported needing more intense stimuli [3]. Abdi et al.’s [8] meta-analysis confirmed a small but significant negative correlation between pornography use and sexual satisfaction (r = −0.10), consistent with desensitization models. The slightly higher rates in our sample may reflect the unique stressors facing Syrian medical students in a conflict-affected society, where pornography may serve as both coping mechanism and source of additional psychological burden.

Despite widespread acknowledgment of pornography’s negative effects on relationships (58.7%), mental health (63.9%), and youth development (78.1%), a majority of participants (67.8%) supported open-access laws, with only 24.1% advocating for restrictions. This apparent contradiction—between perceived harm and policy preference—has been observed in other cultural contexts. Martínez-Fernández et al. [11] noted similar tensions among Spanish health science students, where acknowledgment of harm coexisted with support for individual autonomy. The significant correlation between number of negative effects acknowledged and support for restrictions (ρ = 0.47, p < 0.001) suggests that increased awareness may influence public attitudes toward regulation, particularly when framed around youth protection and mental health advocacy. In the Syrian context, this finding may reflect broader societal ambivalence about the role of state regulation versus personal freedom in the digital domain.

### Limitations

This study has several limitations. First, the cross-sectional design precludes causal inference regarding the relationships identified. Second, self-reported data may be subject to social desirability bias, particularly given the sensitive nature of the topic and conservative Syrian cultural norms. Third, the exclusion of participants with psychiatric disorders limits generalizability to this important subgroup, who may be at elevated risk for problematic use. Fourth, online recruitment may have introduced selection bias toward students with higher digital engagement, potentially inflating prevalence estimates. Fifth, we did not employ a validated scale for problematic pornography use (PPU), instead measuring self-perceived addiction and adverse effects; therefore, comparisons with studies using validated instruments should be interpreted cautiously. Sixth, the cross-cultural adaptation process, while rigorous, cannot guarantee full equivalence of constructs across cultural contexts. Finally, the convenience sampling method may limit representativeness, although the large sample size and diverse institutional representation mitigate this concern to some extent.

### Recommendations

Future research should employ longitudinal designs to establish temporal relationships between pornography consumption, mental health outcomes, and academic performance. Studies specifically examining medical students with psychiatric comorbidities are urgently needed, as this population may be most vulnerable. Validated PPU instruments should be utilized in future Syrian research to enable direct comparison with international prevalence data. Qualitative studies exploring the lived experiences of users—particularly regarding moral incongruence, cessation attempts, and the intersection with conflict-related trauma—would provide deeper contextual understanding. Interventions should be developed that are culturally sensitive, addressing both structural facilitators (privacy, internet access) and psychological correlates (stress, loneliness, trauma) of consumption. Universities should consider establishing non-judgmental support services for students experiencing distress related to pornography use, framed within broader mental health and wellness initiatives rather than moralistic discourses.

## Conclusion

This first investigation of pornography consumption among Syrian medical students reveals high lifetime exposure beginning in early adolescence, comparable to rates reported internationally. Contrary to Western studies, socioeconomic factors—private room access and higher income—emerged as stronger determinants of use than gender. Religious commitment was protective but associated with guilt among consumers. Cessation attempts were common, and withdrawal-like symptoms were frequently reported, resembling behavioral addiction phenomena. Self-perceived addiction was predicted by early exposure, particularly among males. Functional effects included decreased sexual satisfaction and tolerance-like phenomena. Despite widespread acknowledgment of negative effects, most participants supported open-access laws, revealing a tension between perceived harm and policy preferences. These findings underscore the need for culturally tailored, non-judgmental support services addressing both structural facilitators and psychological correlates of consumption among medical students in conflict-affected, conservative societies. Longitudinal studies employing validated instruments are urgently needed to inform evidence-based interventions.
